# Palmitoylethanolamide stimulates phagocytosis of *Escherichia coli* K1 by macrophages and increases the resistance of mice against infections

**DOI:** 10.1186/1742-2094-11-108

**Published:** 2014-06-14

**Authors:** Sandra Redlich, Sandra Ribes, Sandra Schütze, Roland Nau

**Affiliations:** 1 Institute of Neuropathology, University Medical Center Göttingen, 37075 Göttingen, Germany; 2 Department of Geriatrics, Evangelisches Krankenhaus Göttingen-Weende, 37075 Göttingen, Germany

**Keywords:** CNS infection, Palmitoylethanolamide, Phagocytosis, *Escherichia coli*

## Abstract

**Background:**

Palmitoylethanolamide (PEA), an endogenous lipid and a congener of anandamide, possesses a wide range of effects related to metabolic and cellular homeostasis including anti-inflammatory and neuroprotective properties.

**Methods:**

*In vitro*, we studied the ability of macrophages to phagocytose *Escherichia coli* K1 after stimulation with increasing doses of PEA. *In vivo*, wild-type mice were treated with PEA intraperitoneally 12 hours and 30 minutes before infection. Meningoencephalitis or sepsis was induced by intracerebral or intraperitoneal infection with *E. coli* K1.

**Results:**

Stimulation of macrophages with PEA for 30 minutes increased the phagocytosis of *E. coli* K1 without inducing the release of TNFα or CXCL1. Intracellular killing of *E. coli* K1 was higher in PEA-stimulated than in unstimulated peritoneal macrophages and microglial cells. Pre-treatment with PEA significantly increased survival of mice challenged intracerebrally or intraperitoneally with *E. coli* K1. This effect was associated with a decreased production of CXCL1, IL-1β and IL-6 in homogenates of spleen and cerebellum in mice treated with PEA.

**Conclusions:**

Our observations suggest that these protective effects of PEA in mice can increase the resistance to bacterial infections without the hazard of collateral damage by excessive stimulation of phagocytes.

## Background

Palmitoylethanolamide (PEA) is an endogenous lipid that belongs to the family of fatty acid ethanolamines (FAEs) [1]. Under physiological conditions, PEA exists in all cells, tissues and body fluids. It exerts a multitude of physiological functions related to metabolic and cellular homeostasis. The list of properties of PEA has been widely extended since its discovery as an antipyretic compound [2] and, nowadays, PEA is known to act as an anti-inflammatory [3], analgesic [4], and anticonvulsant [5] agent. More recently, PEA has been shown to exert neuroprotective and anti-inflammatory effects *in vivo,* that is in spinal cord and traumatic brain injuries [6] and in neurodegenerative processes such as Parkinson’s [7] or Alzheimer’s disease [8]. For a period of time, PEA was considered to be a cannabinoid receptor 2 (CB2) agonist [9], because several effects were antagonized by the selective CB2 receptor blocker SR144528 [4,10]. Many of its properties have been reported to be dependent on the peroxisome proliferator-activated receptor (PPAR)α [7,8,11,12]. PPARα is up-regulated by PEA in a model of spinal cord injury and causes a decrease of the release of interleukins and tumor necrosis factor-α (TNFα) [7,13]. PEA also appears to act via the transient receptor potential vanilloid-1 (TRPV1) and the orphan G-protein coupled receptor GPR55 [14,15].

PEA is abundant in the central nervous system (CNS) where it is produced by neurons, microglia and astrocytes [16,17]. *In vitro*, PEA enhanced the migration of immortalized murine BV-2 microglial cells without affecting other steps of microglial activation, such as proliferation, microsphere engulfment, and nitric oxide production [18]. In earlier studies our group reported a significant increase of phagocytosis of *Streptococcus pneumonia*e R6 and *Escherichia coli* K1 by primary cultures of microglial cells after PEA treatment [19]. The PEA-mediated effect on microglial bacterial uptake was not accompanied by the concomitant release of proinflammatory cyto-/chemokines observed after microglial activation and known to contribute to neuronal injury [20]. As microglial cells in the CNS, tissue macrophages represent the first line of defense against invading pathogens. PEA can attenuate lipopolysaccharide (LPS)-induced inflammatory responses in the murine macrophage cell line RAW264.7 [21], but there are no data about PEA effects on pathogen uptake by macrophages.

Data on PEA as a prophylactic/therapeutic agent in the management of infections are scarce. In an animal model, oral pre-treatment with PEA increased the resistance of mice to live group A *Streptococcus* challenge as well as to injection of crude preparations of *Shigella dysenteriae* toxin and streptolysin O [22]. In the 1970s, PEA under the brand name Impulsin was tested in six clinical trials and demonstrated its potential at reducing the incidence and severity of acute respiratory infections caused by the influenza virus through a non-specific enhancement of the immune response [23-25]. Since then, no other studies were performed to further investigate the potential of PEA as a prophylaxis or therapy in the management of infections.

Here, we aim to study the effect of exogenous PEA on the phagocytosis of *E. coli* K1 by murine peritoneal macrophages and the protective effect of PEA as a prophylactic agent in experimental murine sepsis and meningitis induced by intraperitoneal (ip) or intracerebral (ic) infection with *E. coli* K1.

## Material and methods

### Preparation of murine peritoneal macrophages

C57/Bl6N mice (eight to twelve weeks old) were anesthetized with a mixture of 100 mg/kg ketamine (Medistar, Holzwickede, Germany) and 10 mg/kg xylazine (Riemser, Greifswald, Germany). Peritoneal lavage was performed with 1 ml sterile PBS using an 18-gauge needle. The peritoneal lavage fluid was collected in a Falcon tube. This preparation step was repeated twice. The collected macrophages were centrifuged for 10 minutes at 900 rpm, and the pellet was suspended in DMEM (Gibco, Karlsruhe, Germany). Cells were counted with a hemocytometer and plated in 96-well plates at a density of 70,000 cells/well.

### Primary mouse microglia cell culture

Primary cultures of microglial cells were prepared from brains of newborn C57/Bl6N mice (p0-p2). After removal of the meninges, cells were mechanically disrupted, treated with trypsin (Sigma-Aldrich, Taufkirchen, Germany) for 10 minutes to isolate the cells, afterwards treated with DNAse (Sigma-Aldrich, Taufkirchen, Germany), centrifuged for 10 minutes at 900 rpm at 4°C and suspended in DMEM supplemented with 10% heat-inactivated fetal calf serum (FCS), 100 U/ml penicillin and 100 μg/ml streptomycin. Cells were plated at a density of two brains per T75 culture flask (Corning Costar, Wiesbaden, Germany) and incubated at 37°C with 5% CO_2_. Microglial cells were isolated by shaking 200x/minute for 30 minutes and plated in 96-well plates at a density of 50,000 cells/well.

### Stimulation of macrophages and microglial cells

Cells were either exposed to 0.1 μg/ml LPS from *E. coli* serotype O26:B6 (Sigma-Aldrich, Taufkirchen, Germany) for 24 hours to induce maximum stimulation, to 0.25 mM palmitic acid (PA, Sigma-Aldrich, Taufkirchen, Germany), to 20 μM fenofibrate (Tocris Bioscience, Bristol, United Kingdom) or to increasing concentrations of PEA (1, 3, 10, 30, 100, 300, 1,000 nM) for 30 minutes or 1 hour. PEA (molecular mass 299.5 Da) was obtained from Tocris Bioscience (Bristol, United Kingdom) and dissolved in 0.01% dimethyl sulfoxide (DMSO) according to the manufacturer’s instructions. We have previously shown that 30 minutes of PEA exposure was more effective at increasing bacterial phagocytosis of microglial cells than 24 hours of PEA stimulation [19]. A control group with DMEM containing 0.01% DMSO was included in all experiments. After stimulation, supernatants were stored at -20°C until measurement of cytokines and chemokines.

### Blocking of PPARα

Cells were exposed 1 hour before the agonists PEA, fenofibrate and palmitic acid, to the PPARα antagonist GW6471 (1 μM) (Tocris Bioscience, Bristol, United Kingdom) for 1 hour to block the PEA target PPARα.

### Bacteria

The *E. coli* strain K1 (serotype O18:K1:H7) originally isolated from the cerebrospinal fluid (CSF) of a child with neonatal meningitis (gift of Dr. Gregor Zysk, Institute of Medical Microbiology, Düsseldorf, Germany) was used *in vitro* and in all experimental infections. Bacteria were grown over night on blood agar plates, harvested in 0.9% saline and stored at -80°C. Frozen aliquots were used for the experiments and diluted with saline to the required bacterial concentration.

### Phagocytosis assay

After stimulation, peritoneal macrophages were exposed to the encapsulated *E. coli* K1 strain for 90 minutes with a ratio of approximately 100 bacteria per phagocyte (6 × 10^6^ colony forming units (CFU)/well). After co-incubation with bacteria, cells were washed with PBS and incubated with DMEM containing gentamicin (final concentration 100 μg/ml; Sigma-Aldrich, Taufkirchen, Germany) for 1 hour to kill extracellular bacteria. Thereafter, cells were washed twice with PBS and lysed with 100 μl of distilled water. The intracellular bacteria were enumerated by quantitative plating of serial dilutions on sheep blood agar plates.

### Intracellular survival assay

LPS- and PEA-stimulated or unstimulated control macrophages or microglial cells were incubated with *E. coli* K1 for 90 minutes. Thereafter, cells were washed with PBS and incubated in DMEM containing gentamicin (100 μg/ml; Sigma-Aldrich, Taufkirchen, Germany) for up to 5 hours to kill extracellular bacteria. At different time points (60, 120, 180, 240, 300 minutes), cells were washed with PBS and lysed with distilled water. The intracellular bacteria were counted by quantitative plating of serial dilutions on sheep blood agar plates.

### *In vivo* experiments

The animal experiments were approved by the Animal Care Committee of the University Medical Center Göttingen, Germany, and by the *Niedersächsisches Landesamt für Verbraucherschutz und Lebensmittelsicherheit (LAVES)*, Braunschweig, Lower Saxony, Germany. Twelve-week-old C57Bl/6N mice were used in all experiments. Animals were weighed and scored daily (0, no apparent behavioural abnormality; 1, moderate lethargy; 2, severe lethargy; 3, unable to walk; 4, dead).

Animals were treated ip 12 hours and 30 minutes before infection. The PEA-treated group received 0.1 mg/kg PEA in 250 μl of 0.9% NaCl containing 0.3% DMSO. The control group was treated with 250 μl of 0.9% NaCl containing 0.3% DMSO (saline-treated group). In survival experiments, the systemic infection was induced by ip injection of 1 × 10^5^ CFU *E. coli* K1/mouse in 250 μl of 0.9% NaCl. Meningoencephalitis was induced by injection of 1 × 10^5^ CFU *E. coli* K1/mouse in 10 μl of 0.9% NaCl into the right forebrain close to the meninges. In bacteriological studies, sepsis and meningoencephalitis were induced by ip or ic injection of 3 × 10^6^ CFU *E. coli* K1/mouse in 250 μl 0.9% NaCl or 10 μl 0.9% NaCl, respectively. In survival experiments, animals were monitored over two weeks after infection. In bacteriological studies, animals were sacrificed 24 hours after infection. Mice, which lost more than 20% of their weight or were unable to walk, were sacrificed immediately.

### Tissue preparation

At the end time point, animals were anesthetized with a mixture of 100 mg/kg ketamine (Medistar, Holzwickede, Germany) and 10 mg/kg xylazine (Riemser, Greifswald, Germany), and blood was taken by cardiac puncture, collected in 1.5 ml Eppendorf tubes, stored at 4°C for 30 minutes and then centrifuged at 3,000 g for 10 minutes at 4°C. Serum was stored at -20°C until cyto-/chemokine measurements. Mice were sacrificed by cervical dislocation. The spleen and the brain were removed, and the cerebellum was dissected from the brain stem. One half of the spleen and one half of the cerebellum were homogenized in 500 μl saline. For determination of bacterial concentrations, ten-fold serial dilutions of homogenates and blood in 0.9% NaCl were plated on sheep blood agar plates.

### Cytokine and chemokine measuring

DuoSet ELISA development kits (R&D Systems, Wiesbaden, Germany) were used for the cytokine measurements. CXCL1 (chemokine (C-X-C motif) ligand 1; also called KC or GROα) and TNFα concentrations were characterized in the supernatants after macrophage stimulation. CXCL1, IL-1β, IL-6 and IL-10 levels were determined in homogenates of spleen and cerebellum. The limit of detection was 15 pg/ml for CXCL1 and TNFα, and 7.5 pg/ml for IL-1β, IL-6 and IL-10. The color reaction was quantified at 450 nm on a microplate reader (Bio-Rad, Munich, Germany).

### Statistics

Statistical analysis and graphical presentation were performed by GraphPad Prism 5 Software (GraphPad Software, San Diego, CA, USA). When data were not normally distributed, they were expressed as medians with 25%/75% interquartile ranges (IR) and compared by Kruskal-Wallis test followed by Dunn’s multiple comparisons test to correct for repeated testing. Comparisons of individual pairs of not normally distributed data were performed by Mann–Whitney *U*-test.

Normally distributed data were expressed as means ± standard deviations (SD). ANOVA followed by Bonferroni’s multiple comparisons test was used to perform comparisons among more than two groups. Survival curves were compared by log-rank test. For all comparisons, *P* < 0.05 was considered statistically significant.

## Results

### PEA stimulated the phagocytosis of *E. coli* K1 by macrophages

**Figure 1:**
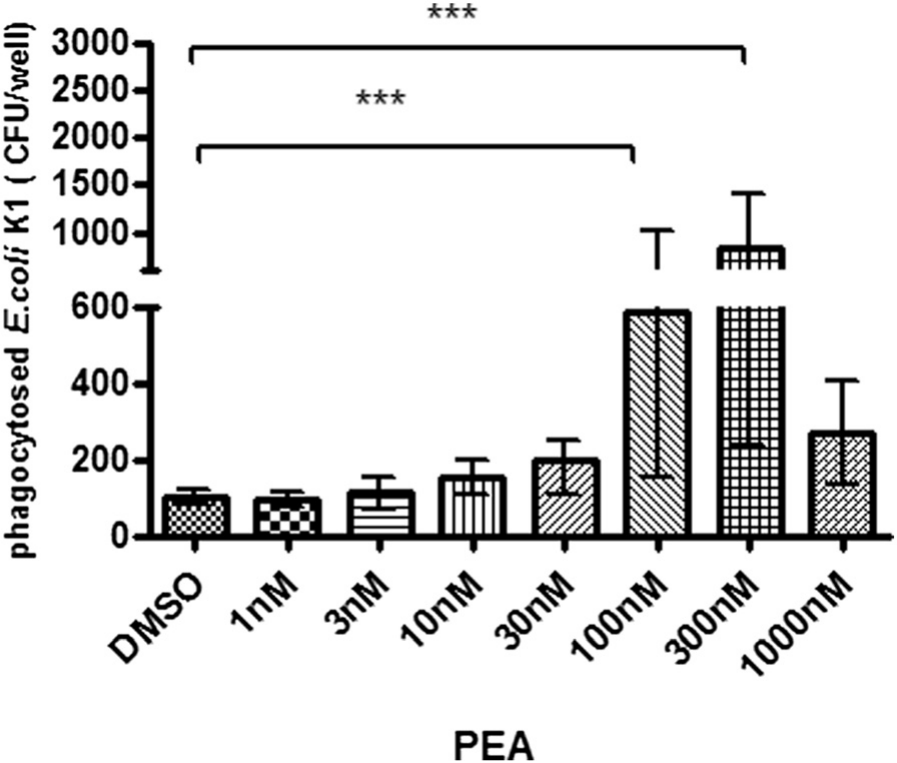
**Phagocytosis of *E. coli *K1 by unstimulated and stimulated macrophages.** Macrophages were either unstimulated (treated with medium containing 0.01% DMSO) or stimulated with PEA for 30 minutes (n ≥ 8 wells/group from two independent experiments). Data are given as means ± SD. Data were analyzed by ANOVA followed by Bonferroni multiple comparisons test (***P < 0.001, **P < 0.01).

The amounts of ingested *E. coli* K1 after 30 minutes of stimulation with increasing doses of PEA are shown in Figure 1. Unstimulated cells (cells stimulated with DMEM containing 0.01% DMSO, DMSO group) ingested bacteria at a low rate (mean ± SD) (96.67 ± 23.38 *E. coli* K1 CFU/well). Pre-stimulation of peritoneal macrophages with different concentrations of PEA for 30 minutes led to an increase of ingested bacteria in a dose-dependent manner. The highest numbers of phagocytosed bacteria were observed after pre-stimulation with 100 and 300 nM PEA (*P* < 0.01 and 0.001 versus DMSO-treated cells, respectively).

### Involvement of PPARα in macrophage stimulation and phagocytosis by PEA

**Figure 2:**
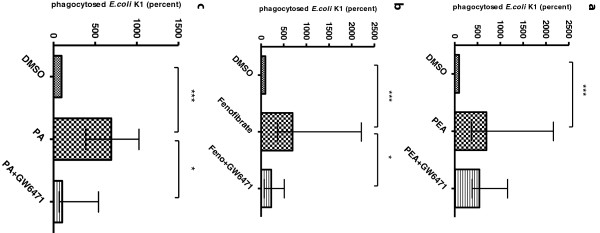
**Involvement of peroxisome proliferator-activated receptor (PPAR)α in macrophage stimulation and phagocytosis of *E. coli *K1 by palmitoylethanolamide (PEA), fenofibrate and palmitic acid (PA).** Macrophages were incubated with the PPARα antagonist GW6471 (1 μM) for 1 hour. After incubation, macrophages were either left unstimulated (treated with medium containing 0.01% DMSO) or stimulated with PEA (300 nM) **(a)**, fenofibrate (20 μM) **(b)** or palmitic acid (PA) (0.25 mM) **(c)** for 1 hour (n ≥ 9 wells/group from two independent experiments). In each experiment, the number of bacteria ingested by the control group (DMSO) was considered to be 100%. Phagocytic rates are presented as the percentage of phagocytosis of the respective control group. Data are given as medians with interquartile ranges and were analyzed by Kruskal-Wallis test followed by Dunn’s correction for repeated testing (***P < 0.001, *P < 0.05).

The numbers of ingested *E. coli* K1 after one hour of PEA, fenofibrate and palmitic acid stimulation are shown in Figure 2. Pre-stimulation of macrophages with PEA, fenofibrate and palmitic acid for one hour induced a significant increase of ingested bacteria (*P* < 0.001 versus DMSO-treated cells). After blocking with the PPARα antagonist GW6471 the amounts of ingested *E. coli* K1 were significantly decreased in the fenofibrate and palmitic acid stimulated group (*P* < 0.05, respectively). GW6471 also tended to reduce the number of phagocytosed bacteria by PEA-stimulated cells, the difference, however, failed to reach statistical significance.

### PEA enhanced intracellular killing of *E. coli* K1 by microglial cells and macrophages

**Figure 3:**
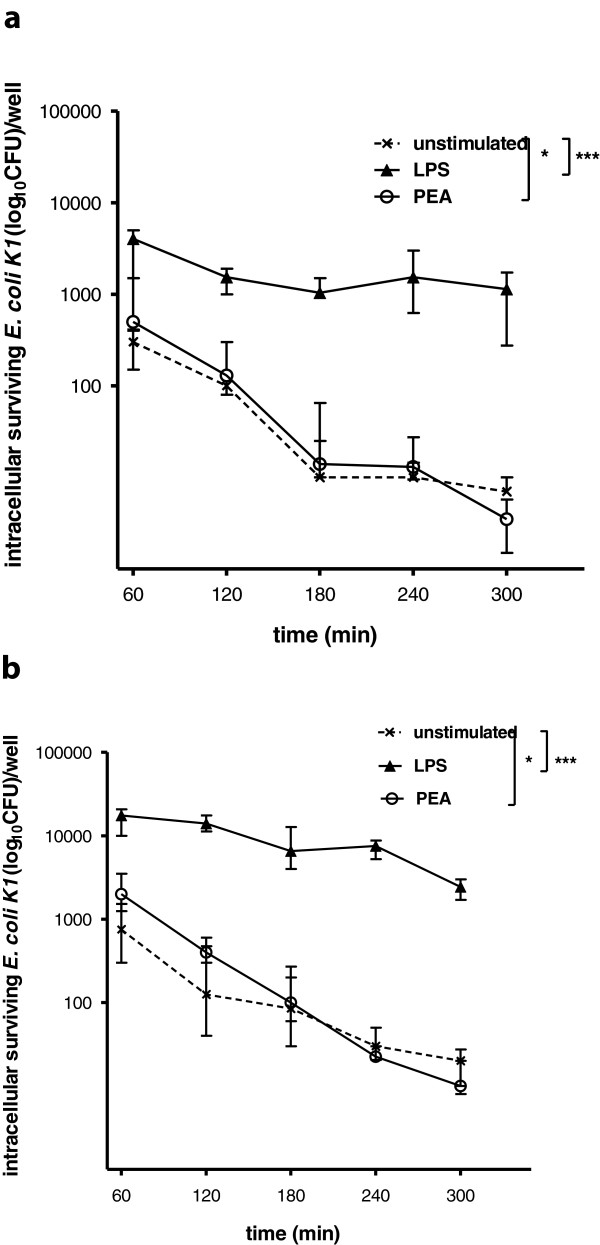
**Intracellular survival of *E. coli *K1 by unstimulated, palmitoylethanolamide (PEA)- and lipopolysaccharide (LPS)-stimulated microglial cells (a) and macrophages (b).** Intracellular survival was expressed as the number of bacteria (median) recovered at the different time points (n ≥ 9 wells/group from two independent experiments **(a)**, n ≥ 12 wells/group from two independent experiments **(b)**). For statistical comparisons the median number of bacteria at zero hours was calculated. Then, the number of bacteria at five hours of each individual well was subtracted from the median number at zero hours, and these differences were compared by Kruskall-Wallis Test followed by Dunn’s correction for repeated testing (***P < 0.001, *P < 0.05).

Stimulation of microglia with LPS and PEA resulted in a significantly higher number of intracellularly killed *E. coli* in comparison to unstimulated cells (*P* < 0.001, *P* < 0.05, respectively) (Figure 3a). The median absolute numbers of intracellularly killed bacteria after 5 hours were 3,000 CFU/well in LPS-treated and 496 CFU/well in PEA-treated groups compared to 293 CFU/well in unstimulated cells.

Pre-stimulation with LPS and PEA induced a higher number of intracellularly killed bacteria by peritoneal macrophages (Figure 3b). The absolute numbers of killed bacteria (medians) were 15,100 CFU/well in LPS- and 1,990 CFU/well in PEA-treated groups compared to 730 CFU/well in unstimulated macrophages (*P* < 0.001, *P* < 0 .05, respectively).

### Prophylaxis with PEA conferred protection against *E. coli* K1 infection

**Figure 4:**
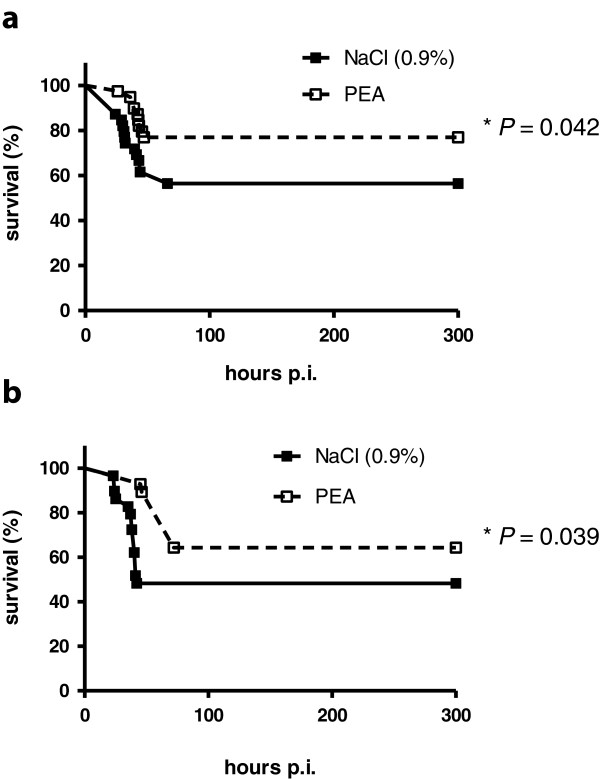
**Prophylaxis with palmitoylethanolamide (PEA) conferred protection against *E. coli *K1 infection.** Kaplan-Meier survival curves of mice pre-treated intraperitoneally with 0.1 mg/kg PEA (250 μl, containing 0.3% DMSO in 0.9% NaCl) 12 hours and 30 minutes before infection (n = 39) and control mice (n = 39) (250 μl, 0.3% DMSO in 0.9% NaCl) after ip infection with *E. coli* K1 **(a)** and of mice pre-treated with 0.1 mg/kg PEA (250 μl containing 0.3% DMSO in 0.9% NaCl) 12 hours and 30 minutes before infection (n = 29) and control mice (250 μl 0.3% DMSO in 0.9% NaCl) (n = 28) after intracerebral infection with *E. coli* K1 **(b)**. The respective groups were compared by log-rank test.

Two ip injections of 0.1 mg/kg PEA protected mice against ip infection with *E. coli* K1 (Figure 4a). Survival at 14 days after infection was 77% (30/39) in PEA-treated mice versus 56% (22/39) in saline-treated mice (*P* = 0.042; log-rank test). Two doses of 0.1 mg/kg of PEA increased the survival of mice infected ic with *E. coli* K1 (Figure 4b). Survival at 14 days after infection was 66% (19/29) in PEA-treated mice versus 46% (13/28) in saline-treated mice (*P* = 0.039; log-rank test).

### Bacterial concentrations in spleen, blood and cerebellum after intracerebral infection

**Figure 5:**
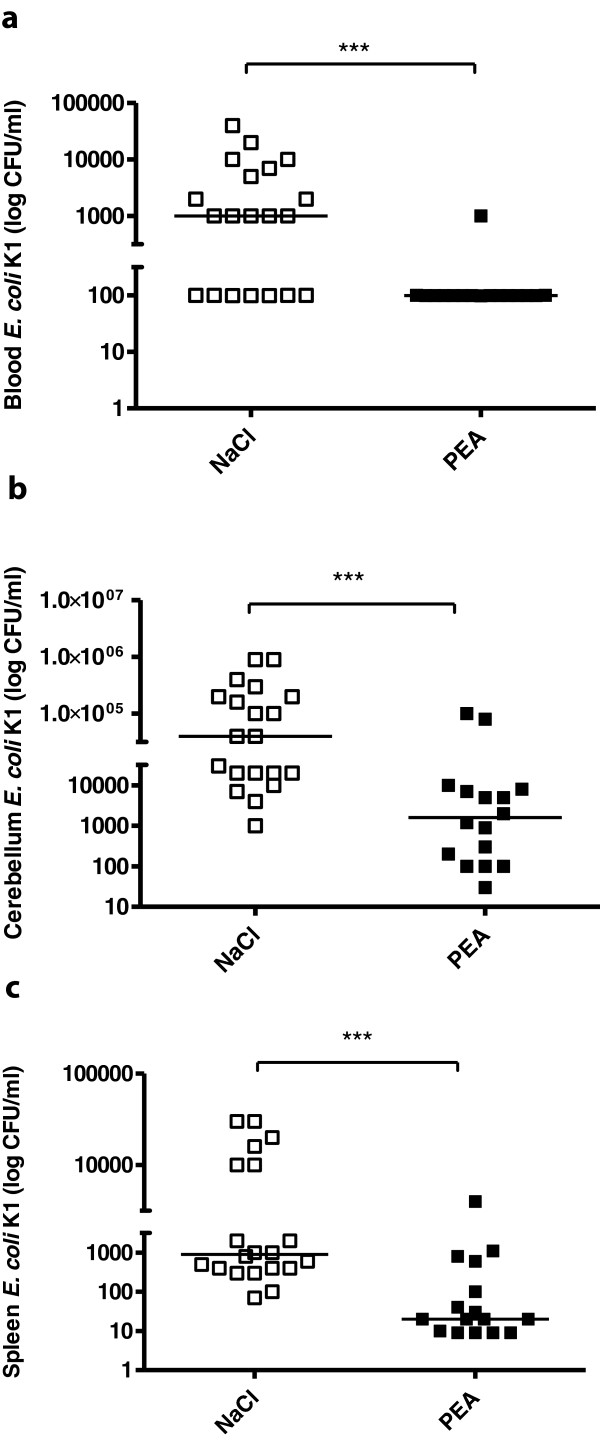
**Bacterial concentrations in spleen, blood and cerebellum after intracerebral infection.** Bacterial concentrations (CFU/ml) in blood **(a)**, cerebellum **(b)** and spleen **(c)** 24 hours after intracerebral *E. coli* infection. Statistical analysis was performed by Mann–Whitney *U*-test. Data are shown as individual measurements and medians (***P < 0.001).

PEA-treated and saline-treated mice were sacrificed 24 hours after ic infection. The bacterial concentration in blood of PEA-treated mice was below the limit of detection in 9 of 10 mice (median below the detection limit) compared to 1 × 10^3^ (1 × 10^2^/6.5 × 10^3^ CFU/ml) in buffer- treated mice (*P* = 0.0001; Mann–Whitney *U*-test) (Figure 5a). The bacterial density in cerebellum of PEA-treated mice was 1.6 × 10^3^ (1.2 × 10^2^/7.7 × 10^3^ CFU/ml) compared to 4 × 10^4^ (2 × 10^4^/2 × 10^5^ CFU/ml) in saline-treated mice (*P* = 0.0001; Mann–Whitney *U*-test) (Figure 5b). Bacterial concentrations in spleen homogenates of PEA-treated mice was 2 × 10^1^ (below the detection limit/4.7 × 10^2^ CFU/ml) compared to 9 × 10^2^ (4 × 10^2^/1 × 10^4^ CFU/ml) in saline-treated mice (*P* = 0.0003; Mann–Whitney *U*-test) (Figure 5c).

### Bacterial concentrations in spleen and blood after intraperitoneal infection

**Figure 6:**
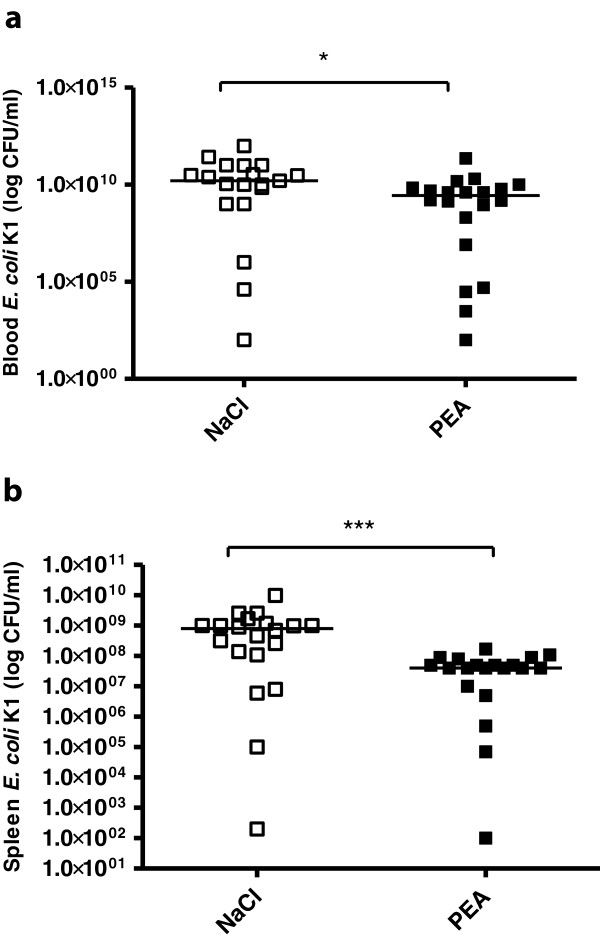
**Bacterial concentrations in blood and spleen after intraperitoneal infection.** Bacterial concentrations (CFU/ml) in blood **(a)** and spleen **(b)** 24 hours after intraperitoneal *E. coli* infection. Statistical analysis was performed by Mann–Whitney *U*-test. Data are shown as individual measurements and medians (***P < 0.001, *P < 0.05).

PEA-treated and saline-treated mice were sacrificed 24 hours after ip infection. The bacterial concentration in blood of PEA-treated mice was 2.8 × 10^9^ (5.6 × 10^7^/6.7 × 10^9^ CFU/ml) compared to 1.6 × 10^10^ (1 × 10^9^/9.9 × 10^10^ CFU/ml) in saline-treated mice (*P* = 0.013; Mann–Whitney *U*-test) (Figure 6a). Bacterial concentrations in the spleens of PEA-treated mice were 4 × 10^7^ (1.7 × 10^7^/7.2 × 10^7^ CFU/ml) compared to 8 × 10^8^ (1.1 × 10^8^/1.1 × 10^9^ CFU/ml) in saline-treated mice (*P* = 0.0005; Mann–Whitney *U*-test) (Figure 6b).

### Cytokine and chemokine measurement

**Figure 7:**
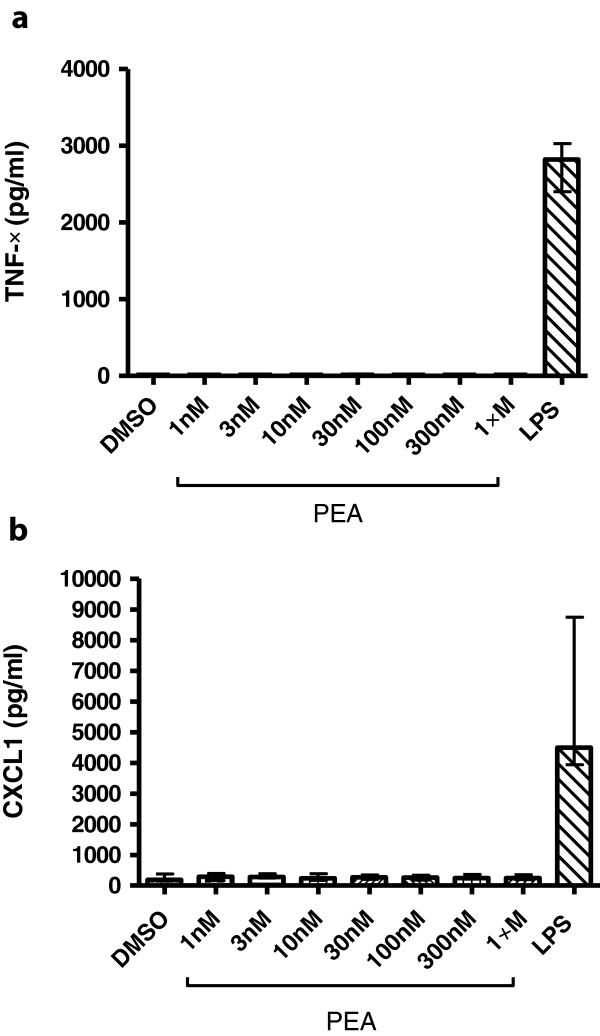
**Cytokine and chemokine release of palmitoylethanolamide (PEA)- and lipopolysaccharide (LPS)-stimulated microglial cells.** TNFα **(a)** and CXCL1 **(b)** concentrations (in pg/ml) in the supernatants of microglial cell cultures after stimulation with different concentrations of PEA for 30 minutes and 0.01 μg/ml LPS for 24 hours. Data are given as medians with interquartile ranges. PEA did not stimulate the release of proinflammatory cytokines and chemokines.

*In vitro*, PEA-mediated stimulation did not induce the release of proinflammatory compounds by peritoneal macrophages (Figure 7). TNFα or CXCL1 levels were comparable between unstimulated (DMSO group) and PEA-stimulated cells. Macrophage stimulation by LPS induced the release of 2,822 (1,564/3,027) pg/ml and 4,497 (3,939/8,742 pg/ml) of TNFα and CXCL1, respectively.

**Figure 8:**
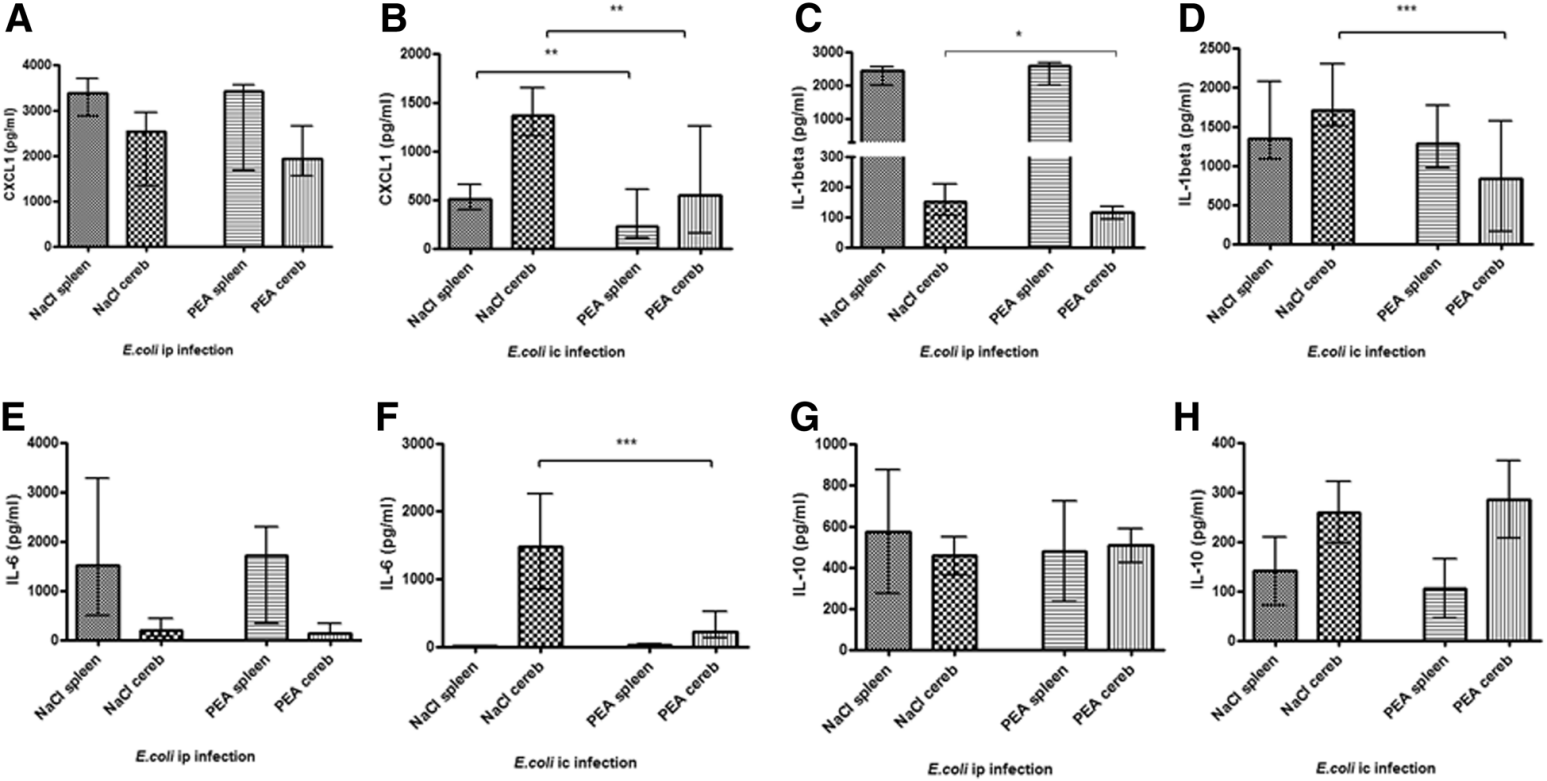
**Cytokine and chemokine concentrations in homogenates of spleen and cerebellum.** CXCL1 **(a/b)**, IL-1β **(c/d)**, IL-6 **(e/f)**, and IL-10 **(g/h)** concentrations (in pg/ml) in the homogenates of spleen and cerebellum (cereb) 24 hours after intraperitoneal or intracerebral infection with *E. coli* K1. Data are given as medians with interquartile ranges. Data were analyzed by Mann–Whitney *U*-test (*P < 0.05, **P < 0.01, ***P < 0.001).

Chemokine and cytokine levels were measured in the homogenates of spleen and cerebellum 24 hours after ic infection with *E. coli* K1 (Figure 8b, d, f, h). CXCL1 levels in both tissues were lower in PEA-treated than in saline-treated mice (*P* = 0.0021 in cerebellum; *P* = 0.0095 in spleen) (Figure 8b). Pre-treatment with PEA reduced IL-1β and IL-6 concentrations in cerebellum compared to the levels determined in the saline-treated group (*P* = 0.0004 and *P* = 0.0001, respectively) (Figure 8d, f). The levels of IL-1β in spleen of PEA-treated mice were lower than in the saline group but the differences did not reach statistical significance. PEA- and saline-treated animals showed similar amounts of IL-10 in both tissues (Figure 8d, h).

Similarly, chemo-/cytokines were quantified 24 hours after ip challenge with *E. coli* K1. In the model of systemic infection, prophylaxis with PEA reduced the levels of IL-1β in cerebellum homogenates (*P* = 0.05) (Figure 8c). CXCL1 levels in cerebellum of PEA-treated mice were also lower than those measured in saline-treated mice but the difference did not reach statistical significance (Figure 8a). The levels of CXCL1, IL-1β and IL-6 in spleen homogenates were similar in PEA- and saline-treated mice (Figure 8a, c, e). PEA and saline-treated animals showed similar amounts of IL-10 in both spleen and cerebellum homogenates (Figure 8g).

## Discussion

Sepsis and meningitis are life-threatening diseases with a high incidence in neonates, infants and in the immunocompromized and older-aged patients in which *E. coli* is one of the most important pathogens [26,27]. Immunocompromized patients, in particular, are susceptible to sepsis and meningitis from a variety of bacteria and fungi. For many of these pathogens, no vaccines are commercially available. Moreover, the effectiveness of vaccination in these patients is often reduced, since most vaccines are less immunogenic in the older age group because of age-related changes in the immune system [28].

For these reasons, infectiologists are highly interested in epitopes shared by several serotypes of the same species or even different species, which are suitable for the simultaneous vaccination against multiple pathogens. Since bacterial DNA, unlike eukaryotic DNA contains a high rate of unmethylated cytosine-guanine (CpG) motifs [29], recent attention has focused on this epitope, which is a ligand of Toll-like receptor (TLR)-9. Stimulation of microglial cells with CpG oligonucleotides increased phagocytosis of *E. coli*, *Streptococcus pneumoniae* and *Cryptococcus neoformans* and intracellular killing of these pathogens by microglial cells [30-32]. In various animal models, CpG ODN pre-treatment conferred protection against a variety of bloodstream and other extracerebral bacterial infections [33-35]. Another epitope shared by many pathogens is muramyl dipeptide (MDP), the smallest peptidoglycan constituent of both Gram-positive and Gram-negative bacteria. It is a ligand of the nucleotide-binding oligomerization domain-like receptor 2 (NOD2), moderately activates microglial cells leading to an increased phagocytosis of bacteria and acts in an additive or synergistic way with TLR agonists [36]. MDP is known to be an adjuvant for vaccination, which induces antigen-specific T and B cell responses, delayed-type hypersensitivity and antibody production. Thirty-five years ago, it was shown that MDP enhances the non-specific immunity to *Klebsiella pneumoniae* infections in adult and newborn mice [37]. Parenteral MDP and two of its analogs protected mice against *Pseudomonas aeruginosa* or *Candida albicans* infections [38]. MDP conjugated with the neoglycoprotein mannosyl human serum albumin (mannose-HSA), in a murine model of visceral leishmaniasis, strongly reduced splenic parasite burden, whereas free MDP at a similar dose had very little effect [39]. Prophylactic treatment with MDP protected mice against *E. coli, Streptococcus pneumoniae, Salmonella typhimurium, Salmonella enteritidis* and *Toxoplasma gondii* infections [40,41]. The synthetic MDP derivate romurtide, given orally or subcutaneously, also enhanced the nonspecific resistance against microbial infections in mice [42].

Prophylactic administration of TLR or NOD ligands can lead to an unspecific inflammatory state characterized by the release of proinflammatory cytokines by immune cells and elevated cytokine concentrations in the systemic circulation. This may be a severe disadvantage for an organism attempting to combat an infection, because activated phagocytes are not only able to efficiently eliminate pathogens, but can also acutely damage host tissue or lead to chronic autoimmune diseases [43,44]. This phenomenon is most devastating in the CNS [45,46]. There, it probably contributes to neuronal and axonal injury in the course of meningitis, encephalitis and septic encephalopathy [47]. It probably also is the pathophysiological basis of the deterioration of patients with neurodegenerative diseases during infections. The functional outcome of TLR-induced activation of microglia in the CNS depends on a subtle balance between protective and harmful effects [48-50]. For this reason, activation of the TLR or NOD system aiming at increasing the resistance to infections bears the risk of inducing collateral damage to the vessels, the nervous system or other organs.

PEA, an endogenous compound found in most mammalian tissues, has well-known anti-inflammatory, neuroprotective and analgesic properties [1]. Moreover, oral PEA pre-treatment (optimum dose 50 mg/kg/day for 12 consecutive days) increased the resistance of mice against challenge with *Shigella dysenteriae* toxin and streptolysin O, but also against intravenous infection with live group A streptococci [23]. Unlike TLR or NOD agonists, PEA does not induce the release of TNFα, IL-6 and CXCL1 by microglial cells [19]. It therefore cannot be considered a mere immunostimulant, but a true immunomodulator [51].

PEA is known for its anti-inflammatory activity and effect on interleukins. PEA was shown to attenuate the factors of intestinal injury during inflammation and to inhibit proinflammatory cytokine production (TNFα, IL-1β), adhesion molecules (ICAM-1, P-selectin) expression, and NF-κB expression [52].

Many effects of PEA have been shown to be dependent on the peroxisome proliferator-activated receptor-α (PPARα) [11,12]. In our study, the phagocytic rate in PEA-stimulated macrophages was not as strongly decreased by the PPARα inhibitor GW6471 as in macrophages stimulated by the PPARα agonists fenofibrate and palmitic acid. This suggests that the properties of PEA do not only depend on the stimulation of PPARα. In the present study, PEA administered ip did not only act at the site of infection, but also protected against injection of bacteria into the CNS. This compares well with the protection against intravenous infection by oral PEA administration [23]. Since the immune system of the CNS is separated from the systemic circulation by the blood–brain and blood-CSF barrier, the CSF and by the glia limitans composed of astrocytic foot processes and a parenchymal basement membrane [53], our results suggest that PEA treatment influences the immune defense of the whole organism including the deep compartments. Early clinical trials with PEA in the 1970s, at that time under the trade name of Impulsin, demonstrated its potential of reducing the incidence and severity of acute respiratory infections [25]. Unfortunately, since then, no other studies focusing on PEA as a prophylaxis or as an adjuvant therapy in the management of infections have been published. Concerning its safety, more than 3,600 patients have been successfully treated with PEA, with no adverse effects reported in any of the trials [51,54].

In conclusion, PEA appears to increase the resistance of animals and humans against bacterial infections without inducing a chronic inflammatory state. Its efficacy should be studied in immunocompromized animals and with a broader range of pathogens. Because of the apparently low rate of adverse effects, PEA is a promising compound for a clinical trial in patients at a high risk of developing life-threatening infections.

## Abbreviations

CB2: cannabinoid receptor 2; CFU: colony forming units; CNS: central nervous system; CSF: cerebrospinal fluid; CXCL1: chemokine (C-X-C motif) ligand 1; DMEM: Dulbecco’s modified Eagle’s medium; DMSO: dimethyl sulfoxide; ELISA: enzyme-linked immunosorbent assay; FAEs: fatty acid ethanolamines; FCS: fetal calf serum; GRO: growth-regulated oncogene; ic: intracerebral; IL: interleukin; ip: intraperitoneal; LPS: lipopolysaccharide; MDP: muramyl dipeptide; NF-κB: nuclear factor kappa beta; NOD2: nucleotide-binding oligomerization domain-like receptor 2; PA: palmitic acid; PBS: phosphate-buffered saline; PEA: palmitoylethanolamide; PPAR: peroxisome proliferator-activated receptor; TLR: Toll-like receptor; TNFα: tumor necrosis factor alpha; TRPV1: transient receptor potential vanilloid-1.

## Competing interests

The authors declare that they have no competing interests.

## Authors’ contribution

SRe performed the experiments and wrote the manuscript, SR, SS and RN planned and designed the study. All authors read and approved the final version of the manuscript.
